# The Buffering Effect of Health Care Provider Video Biographies When Viewed in Combination With Negative Reviews: “You Can’t Fake Nice”

**DOI:** 10.2196/16635

**Published:** 2020-04-14

**Authors:** Evan K Perrault, Grace M Hildenbrand

**Affiliations:** 1 Brian Lamb School of Communication Purdue University West Lafayette, IN United States

**Keywords:** patient ratings, health care providers, video, biographies, expectancy violations, thin slice

## Abstract

**Background:**

Patients seek information from numerous sources before choosing a primary care provider; two of the most popular sources are providers’ own online biographies and patient rating websites. However, prior research has generally only examined how these sources influence patients’ decisions in isolation.

**Objective:**

This study aimed to determine how primary care providers’ online biographies and online patient ratings interact to affect patients’ decision making, especially in the face of negative reviews.

**Methods:**

An 8-condition online experiment (n=866) was conducted, manipulating patient ratings and the timing of viewing a provider’s online biographical video (pre- or postrating viewing).

**Results:**

When participants were shown a short video introduction of a provider after reading predominantly negative reviews a positive expectancy violation occurred, which was also related to more positive perceptions of the provider. When exposed to all negative reviews, 43% of participants indicated they would still choose to make an appointment with the provider, with many indicating that the video provided the evidence needed to help make up their own minds.

**Conclusions:**

These findings are especially relevant to health care organizations seeking to combat a recent rise in fake patient reviews. Providing patients with realistic expectations of the care that clinicians can offer via their own online biographical videos can help counteract negative patient comments online.

## Introduction

### Background

Selecting a new primary care provider, “one of the most important health-related decisions a patient makes” [1], can be a daunting task with numerous qualities to consider. For example, can I easily get an appointment; will the provider treat me with respect; will I feel comfortable communicating with this provider? As a result of governments’ and health care organizations’ directives to provide greater levels of patient-centered care (PCC), patients arguably have more information at their fingertips than ever before to help make this decision.

Outside of recommendations from others, one source that prospective patients use to gain information about providers is through biographies on health care systems’ websites [2]. Other sources some patients consult are physician online ratings websites, in which patients give reviews in the form of numerical ratings and include narrative comments about their experiences with providers [3].

Given the importance of selecting a provider, patients likely consult information from multiple sources. However, previous research has generally examined patients’ decision-making processes and their perceptions of providers based on visiting only one type of information source. For example, Perrault and Silk [4,5] examined prospective patients’ attitudes when experimentally manipulating content within providers’ online biographies. Others have solely investigated the effect of providers’ online ratings on patients’ beliefs and decision making [6-8]. What happens when prospective patients examine multiple sources of information to make their decisions? If researchers continue to only study individual sources of information in isolation, a complete understanding of the impact that the totality of this information can offer will never be fully realized.

Therefore, guided by expectancy violations theory [9,10] and the concept of thin slicing [11], this research sought to understand how both the information provided by providers’ own online biographies and that offered on rating websites might interact in influencing prospective patients’ perceptions of a primary care provider and patients’ decision making. The results could also provide important information to health care organizations on the strong influence their providers’ own online biographies may have, especially in the face of negative reviews.

### The Growing Information About Providers

Providing PCC is becoming a growing necessity in the health care industry. In the near future, some degree of provider reimbursements is going to be tied to the Agency for Healthcare Research and Quality’s (AHRQ’s) Consumer Assessment of Healthcare Providers and Systems (CAHPS) survey measures [12], an assessment that among other things measures patient-centered experiences (eg, ease of access to health care services and provider communication). A part of providing greater levels of PCC is offering patients information to enable them to be more informed decision makers, especially in helping them choose a provider or practice that is most likely to meet their individualized needs [13]. The industry is therefore seeing increased innovation and research into the development and improvement of information available to patients to help them make decisions on choosing providers. Two of these information sources are providers’ own online biographies and third-party rating websites.

#### Online Biographies

In a recent survey of almost 4000 people, Perrault and Hildenbrand [2] found that the most popular source from which prospective patients sought information about providers was through online biographies provided by health care organizations. In an industry that is increasingly becoming more consumer-centric [14], with systems competing for patients, there has been greater attention paid toward finding ways to help health care organizations improve outward-facing communication about their providers to prospective patients. After all, the information provided by health care systems on their own websites about providers is under their complete and direct control [15]. Although patients desire the technical expertise of providers, which is often displayed within providers’ online biographies through the articulation of degrees and fellowships [16], patients strongly care about the communication qualities of providers. Specific qualities valued by patients include a provider engaging in active listening, being friendly, explaining information in an understandable way, and having a good bedside manner [17,18]. These types of qualities can usually be conveyed within online biographies through philosophy of care statements or even through video introductions of providers [5,19], and it is therefore likely why these types of communicative qualities are also usually displayed on rating websites.

#### Rating Websites

There are a growing number of online provider rating websites for patients to choose from when seeking information about prospective providers [3,20]. Most often these rating sites consist of quantitative ratings and narrative comments in which patients rate or describe personal experiences with particular providers [3]. These websites request the patient’s feedback in categories such as physician’s knowledge, timeliness, and interpersonal skills [21].

However, although large numbers of patients do seek information from these rating websites [3,22], most do so with caution. “Americans do not seem to put much stock in overall rating systems of doctors or other care providers” [17]. Only about 10% of Americans “completely”, or “trust very much”, the provider quality information provided by ratings websites, and only about 30% would trust quality ratings from patients who are surveyed anonymously about the quality of their care [17]. In addition, most ratings on rating websites tend to be positive [6], indicating that patients who consult these sites may not be receiving a fully representative picture of other patients’ experiences [23]. In other words, patients who solely make decisions based on rating websites may choose providers who violate the patients’ expectations.

### Expectancy Violations Theory

Expectancy violations theory is rooted in the belief that everyone has or develops expectations about what a future interaction with someone should and will be like [10]. In the case of seeking a new health care provider, prospective patients could develop these expectations by reading other patients’ comments on rating websites discussing their experiences with a specific provider. Even though prospective patients do not place much confidence in patient rating websites to select providers, about two-thirds of those surveyed in a nationally representative sample indicated that patients’ ratings of providers’ communication are an important factor [17]. Patients are less likely to visit doctors when they are rated negatively, and this is especially the case when negative reviews are shown before positive reviews [7]. In other words, once a negative expectation of the provider is set, that negative expectation may persist and influence a patient’s ultimate decision to not visit the provider.

However, expectancy violations theory also posits that a person may modify his or her perceptions of a target when the target’s actual communication runs counter to what is expected [9,10]. As Burgoon and LePoire [24] found, negatively induced preinteractional expectancies about a target could be overcome after having a pleasant conversation with that target: “To the extent that uncertainty is introduced by mixed expectancies...perceivers should be motivated instead to attend more to the actual behavioral evidence” [24]. One way that health care organizations are beginning to provide this behavioral evidence to prospective patients is through the development of short video introductions of providers to place within online biographies [19]. Therefore, if given a short video introduction of a provider showcasing positive communication skills, that video may be able to override the initial negative expectancies induced by negative reviews.

Hypothesis 1: Participants who first view predominantly negative reviews (all negative or two-third negative) will have more significant expectancy violations of the provider after subsequently viewing a short video of the provider than those exposed to predominantly positive reviews.

### Video Biographies as Thin Slices

People’s ability to accurately predict attributes of others after only viewing short video clips has been termed *thin slicing* [11]. Thin-slice research has found that participants are able to make accurate judgments of targets from as little as 6-second silent videos [25]. Others have found that attributes such as sexual orientation can be predicted from as little as 10-second clips [26], the level of altruism from 20-second clips [27], and personality traits from 30-second clips [28].

In addition, in watching short video biographies of providers, prospective patients are able to actually see the providers’ personality traits, thereby helping them better predict how the provider might interact in a consultation [19]. For example, one participant in Perrault’s [19] study of provider videos indicated the videos to which she was exposed “helped me see if I would feel comfortable with that person.”

Therefore, viewing a video after reading predominantly negative reviews (ie, a positive violation) might actually repair the initial negative perceptions prospective patients had about the provider such as provider liking, trustworthiness, expertise, anticipated patient satisfaction, and anticipated medical care quality—qualities that prior research finds are important to patients [29-31]. Conversely, viewing a video before reading negative comments also might provide a protective effect, lessening any negative impact those comments could have had if simply viewed in isolation. Therefore, we hypothesized the following:

Hypothesis 2: A significant interaction between the viewing order of provider content (video and patient reviews) and the valence of the reviews viewed (all positive, two-third positive, all negative, and two-third negative) on the dependent variables of provider liking, trustworthiness, expertise, anticipated patient satisfaction, and anticipated quality of medical care will be observed.

### Choosing a Provider

After considering all the information available, patients ultimately have to make a choice [32]. To the best of the authors’ knowledge, this is the first study to have participants view both health care system–controlled biographical information of the provider and third-party patient ratings before making decisions. Therefore, a series of research questions (RQs) were posed:

- RQ1: How will the information viewing condition be related to provider selection?
- RQ2: What information influences people’s decision the most regarding whether or not they would want to select the provider?

Finally, given that we predicted that an expectancy violation will occur when people who are exposed to negative reviews subsequently see the provider’s video, we believed that there will be some people who will choose to visit the provider even in the face of all negative reviews. Therefore, we are curious how people will explain their decisions when provided all negative reviews with the following RQ:

- RQ3: When exposed to a condition containing all negative reviews, what reasons do people provide for wanting, or not wanting, to choose to visit the provider?

## Methods

This study took the form of a 4 (provider ratings: all positive, two-third positive, all negative, two-third negative) x 2 (viewing order of provider content) mixed design experiment, where the provider ratings (all positive, two-third positive, all negative, and two-third negative) was the between-subjects factor and the viewing order (video first–reviews second vs reviews first–video second) was the within-subjects factor.

### Procedures and Scenario

Upon consenting and indicating that they were using a device in which they could view the video and listen to the audio, participants were recruited into the study. Participants were asked to imagine themselves as patients who had recently moved across the country for a new job and had fallen ill. After a few days of rest and not feeling any better, they decided it was time to go to a health care provider. They went online to look for a nearby clinic and provider who fit with their health insurance. One half of the participants were told that their first stop online was the health care provider’s own website where a video of the provider they were considering could be found. The other half were told that their first stop online was a popular website where patients’ ratings of health care providers existed, and they looked up the ratings of a provider they were considering. At the end of the study, participants were asked to rate on a one-item measure (1=strongly disagree and 7=strongly agree) on how realistic they thought the scenario regarding selecting a new provider seemed. A one-sample *t* test revealed a mean score significantly above the scale’s midpoint (mean 5.99, SD 0.94; t_852_=61.73; *P*<.001), indicating that participants thought this scenario was realistic.

### Provider Content—Experimental Manipulations

#### The Video

Participants were exposed to a 68-second video of a nurse practitioner who was interviewed discussing her philosophy of care, what a normal consultation with her is like, and what she likes to do when she is not at the clinic. The practitioner was shot in an interview style, with her head and shoulders in the frame. The majority of the interview footage was covered up with B-roll of the provider actually interacting with a patient. For example, a participant could see the provider actually asking questions to the patient, performing a brief examination, and then discussing treatment options with the patient. This B-roll of the provider was included within the video as prior research indicated that prospective patients would like B-roll included in video introductions [19]. The video was produced and edited by the first author who is also a former television reporter. To try and ensure that the participants actually viewed the video, participants were not able to continue with the survey until 68 seconds (the length of the video) had elapsed. Underneath this video was a brief biography of the provider that only provided her name, photo, specialty, and educational credentials (see Multimedia Appendix 1). This basic content is usually provided within most online provider biographies [16].

#### Likeability Induction of the Provider

All participants who viewed the video first (n=413) rated the provider as likable (see the Measures section). A one-sample two-tailed *t* test found responses significantly above the midpoint of the scale (mean 6.38, SD 0.75; t_412_=64.52; *P*<.001). Therefore, this video succeeded at showing participants a provider who is initially perceived as likable by individuals without any information to the contrary.

#### The Reviews

Reviews of the provider were developed by simulating the page of a provider rating website (see Multimedia Appendix 2). Patients’ comments were developed by the second author who viewed hundreds of real patients’ comments on numerous rating websites to create the most realistic comments possible. All comments were solely focused on the communication between that patient and the provider. This is because patients’ ratings of providers’ communication play an important role in prospective patients’ decision making [17,33]. In addition, a majority of clinicians and patients agree that providers should not be evaluated by patients on the clinicians’ technical skills but do agree that patients have the knowledge to evaluate the clinicians’ communication skills [34].

Each review had 3 patient comments, followed by a 5-point star rating. Four sets of patient comments were developed to which a participant could have been randomly assigned: all 3 positive comments, all 3 negative comments, 2 positive comments out of 3 comments (middle comment negative), and 2 negative comments out of 3 comments (middle comment positive). Positive comments were all given five stars, and negative comments were all given one star. Negative comments were an exact opposite translation of the positive comments. For example, if a positive comment said that the provider “always pays attention to me,” the negative version of that same comment said that the provider “never pays attention to me.”

#### Viewing Order

To test this study’s hypotheses and RQs, the viewing order of the video and reviews was also randomly assigned. Half of the participants were randomly assigned to view the video first and the other half randomly assigned to view the patients’ reviews first. After viewing each portion of the provider content, participants completed a series of survey measures.

### Measures

Unless otherwise noted, all variables were measured at two timepoints, once after each exposure to the video and patient reviews.

#### Liking

Provider liking was measured with four items adapted from a study by Jayanti and Whipple [35]. Participants rated their level of agreement on a 7-point Likert scale (1=strongly disagree and 7=strongly agree) with the following statements: This provider seems likable, pleasant, friendly, like a nice person (alpha=.992 and alpha=.984 for the first and second times, respectively).

#### Trust

Provider trust was measured with six 7-point, semantic differential items adapted from source credibility scales of McCroskey and Teven [36] and Ohanian [37]. Participants were asked to rate how dishonest-honest, undependable-dependable, unreliable-reliable, insincere-sincere, untrustworthy-trustworthy, and phony-genuine they perceived the provider to be (alpha=.980 and alpha=.977 for the first and second times, respectively). Higher scores indicated greater levels of trust.

#### Expertise

Expertise was measured with six 7-point, semantic differential items also adapted from the source credibility scales of McCroskey and Teven [36] and Ohanian [37]. Participants rated how they believed the provider to be an expert/not an expert, inexperienced/experienced, incompetent/competent, unqualified/qualified, unskilled/skilled, and stupid/smart (alpha=.971 and alpha=.969 for the first and second times, respectively). Higher scores indicated greater levels of expertise.

#### Anticipated Patient Satisfaction

Anticipated patient satisfaction was measured with three 7-point, semantic differential items from Richmond et al’s [38] satisfaction with the physician scale. Participants were asked to indicate how displeased-pleased, dissatisfied-satisfied, uncomfortable-comfortable they would be with their visit (alpha=.987 and alpha=.974 for the first and second times, respectively). Higher scores indicated greater satisfaction.

#### Anticipated Quality of Medical Care

The anticipated quality of medical care was measured using four items adapted from Richmond et al’s [38] perceived quality of medical care measure. Participants were asked to indicate where they would fall along the 7-point continuum for the following word pairs regarding the kind of medical care they would obtain from the provider: impersonal-personal, uncaring-caring, unconcerned-concerned, and unsatisfactory-satisfactory (alpha=.984 and alpha=.977 for the first and second times, respectively). Higher scores indicated greater perceptions of care quality.

#### Expectancy Violation

Expectancy violation was measured only once, after participants were exposed to the video. Expectancy violation was measured using three items adapted from a study by Klingle and Burgoon [39]. Participants rated on a 7-point Likert scale (1=strongly disagree and 7=strongly agree) their level of agreement to the following prompt and statements: Based on the video you just saw of the provider, please rate your level of agreement with each statement: Kris communicated in a way that I expected; Kris’ communication style is what I anticipated it would be; I expected that Kris would interact with the patient in the way she did (alpha=.960). Lower scores indicated a greater expectancy violation.

#### Decision Making

After viewing both pieces of provider content (video and reviews), participants were asked to indicate (yes/no) their decision regarding the following question: “Based on all the information that you saw, would you decide to make a medical appointment with Kris?”

#### Influence of Content

Participants were then asked to indicate via a closed-ended response about what information influenced them the most regarding whether or not they would make an appointment with the provider. Participants could select from the following three options: provider’s video and biography, patients’ ratings of the provider, or both the video/biography and patients’ ratings.

#### Rationale for Decision

Participants were then asked to respond to an open-ended question inviting them to indicate why they would or would not choose to make an appointment with the provider.

### Participants and Data Cleaning

Participants were recruited utilizing Amazon Mechanical Turk (Seattle, WA) in October 2018 and paid US $1 for participating. Participants were only recruited from the United States. To ensure integrity of the data, multiple procedures were utilized to clean the dataset. An initial captcha item was used to ensure humans, and not machines, were the actual participants. Initially, 1716 surveys were completed. Utilizing procedures outlined by Dennis et al [40] regarding how to identify and remove participants who circumvent initial screening methods (eg, using server farms to circumvent country of residence), the following methods were used. Responses that originated from duplicate GPS coordinates were initially removed (n=301); next, those originating from duplicate internet protocol addresses were also removed (n=105). Three people were removed who did not watch the video, and 138 were removed for not completing more than half of the study’s questions. An additional 87 people were removed for indicating that they had previously seen the provider, 96 people who worked in the health care industry were removed, and 109 people who took less than 8 min to complete the study (an approximate time to reasonably view all stimuli and answer questions) were removed. Finally, 11 participants were removed who did not logically answer the open-ended question asking why they would (or would not) choose to make an appointment, eg, “for meet her” and “I make.” This left a final participant pool of 866 valid responses.

### Demographics

The average age of participants was 39.2 years (SD 12.6; range 18-82). A little more than half of the participants identified as female (n=493). Most participants (626/866, 72.3%) identified as Caucasian, followed by African American (92/866, 10.6%), Hispanic (63/866, 7.3%), Asian (56/866, 6.5%), Native American (8/866, 0.9%), Pacific Islander (2/866, 0.2%), and other (16/866, 1.8%). Participants came from all states except North Dakota. Six participants reported their highest level of education as never completing high school; other participants reported completing a high school diploma/general education diploma (225/866, 26.0%), 2-year college degree (182/866, 21.0%), 4-year degree (336/866, 38.8%), and an advanced college degree (114/866, 13.2%).

## Results

### Hypothesis 1

Hypothesis 1 predicted that participants who first viewed predominantly negative reviews of the provider (ie, all negative or two-third negative) would have a more significant expectancy violation of the provider after subsequently viewing the provider’s video than those who were initially exposed to predominantly positive reviews. To test this hypothesis, a one-way analysis of variance (ANOVA) was conducted where the provider’s review condition was the independent variable and expectancy violation was the dependent variable. The analyses revealed a significant finding: *F*_7, 858_=93.11 and *P*<.001. Post hoc comparisons using the Tukey honestly significant difference test at *P*<.05 showed that those who initially saw all negative reviews had the most significant expectancy violation after viewing the video (mean 3.34, SD 1.76), followed by those who saw two negative comments and then the video (mean 4.14, SD 1.51). All the other six conditions were not statistically different from one another (see Table 1). Therefore, hypothesis 1 was supported. Those who viewed predominantly negative reviews of the provider and then viewed her video had the most significant expectancy violations.

**Table 1:** Expectancy violations by condition.

Dependent variable	Condition, mean (SD)								*F* test (*df*)	*P* value
	Video first,...then reviews				...Reviews first, then video					
	All positive (n=105)	Two-third positive (n=101)	All negative (n=106)	Two-third negative (n=101)	All positive (n=126)	Two-third positive (n=100)	All negative (n=114)	Two-third negative (n=113)		

Expectancy violation	5.72^a^ (0.91)	5.85^a^ (0.97)	5.87^a^ (0.95)	5.88^a^ (0.93)	6.26^a^ (0.78)	5.84^a^ (0.94)	3.34^b^ (1.76)	4.14^c^ (1.51)	93.11 (7, 858)	<.001

^a-c^Means with different superscripts differ at *P*<.05 using the Tukey honestly significant difference test. Expectancy violation was only measured after viewing the video of the provider.

### Hypothesis 2

**Figure 1:**
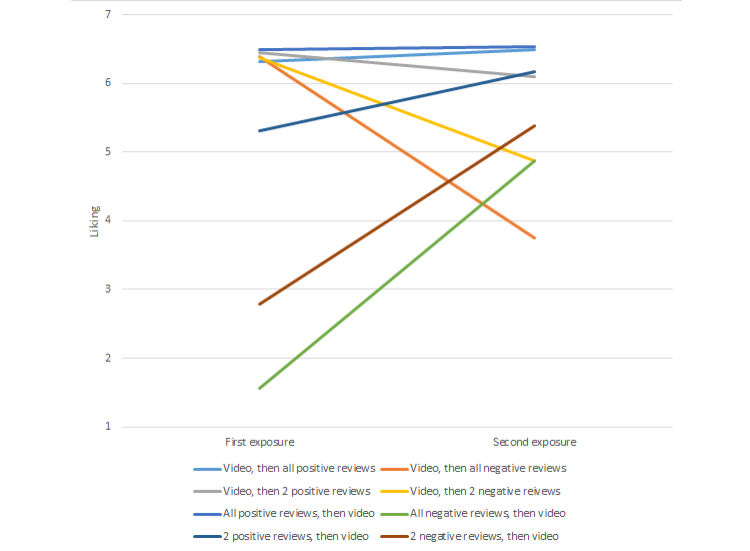
Interaction effect between content viewing order and valence of reviews.

Hypothesis 2 predicted that a significant interaction would arise between the viewing order of the provider content and the valence of the reviews viewed on the dependent variables of provider liking, trustworthiness, expertise, anticipated patient satisfaction, and quality of medical care. A series of mixed ANOVA for each dependent variable was conducted, where the valence of reviews was the between-subjects factor and the viewing order of the provider content (video/reviews either first/second) was the within-subjects factor. The analyses revealed significant interactions for all five dependent variables in the same pattern (see Table 2 for descriptive data and analyses and Figure 1 for a visual depiction of one of the interactions). In general, those who viewed predominantly negative comments first saw their attitudes toward the provider increase significantly after viewing the video of the provider. In fact, in every instance these participants’ attitudes increased to a point significantly above the midpoint of the 7-point scale according to one-sample *t* tests. Conversely, participants who viewed the video first and then were exposed to predominantly negative reviews saw their attitudes significantly decrease. However, in none of these instances did subsequent attitudes decrease significantly below the midpoint of the 7-point scale according to one-sample *t* tests. Therefore, although the videos were not able to hold participants’ initial positive attitudes stable in the face of subsequently viewing predominantly negative reviews, these participants did not ultimately hold negative attitudes toward the provider, or the care that could be provided, after viewing the negative reviews.

**Table 2:** Descriptive statistics and mixed analysis of variance results.

Dependent variables and exposure		Condition, mean (SD)								Condition×exposure		Partial η^2^
		Video first, then...reviews				...Reviews first, then video				*F* test (*df*)	*P* value	
		All positive (n=105)	Two-third positive (n=101)	All negative (n=106)	Two-third negative (n=101)	All positive (n=126)	Two-third positive (n=100)	All negative (n=114)	Two-third negative (n=113)			
**Liking**										296.64 (7, 858)	<.001	0.708
	First	6.32 (0.78)	6.44 (0.78)	6.39 (0.75)	6.37 (0.69)	6.49 (0.59)	5.31 (0.78)	1.56 (0.87)	2.79 (0.99)			
	Second	6.49 (0.72)	6.10 (0.75)	3.76 (1.81)	4.88 (1.34)	6.54 (0.75)	6.17 (0.69)	4.87 (1.27)	5.39 (1.04)			
**Trust**										179.79 (7, 858)	<.001	0.595
	First	6.31 (0.84)	6.52 (0.70)	6.44 (0.92)	6.47 (0.78)	6.36 (0.79)	5.64 (0.87)	3.05 (1.43)	3.81 (1.19)			
	Second	6.47 (0.82)	6.26 (0.80)	4.17 (1.83)	5.14 (1.39)	6.59 (0.69)	6.32 (0.79)	5.16 (1.23)	5.57 (1.09)			
**Expertise**										101.61 (7, 858)	<.001	0.453
	First	6.21 (0.99)	6.30 (0.85)	6.24 (0.97)	6.33 (0.76)	6.25 (0.81)	5.79 (0.95)	3.69 (1.47)	4.52 (1.14)			
	Second	6.35 (0.88)	6.22 (0.83)	4.89 (1.61)	5.45 (1.23)	6.46 (0.73)	6.24 (0.96)	5.49 (1.16)	5.79 (1.02)			
**Patient satisfaction**										249.18 (7, 856)	<.001	0.671
	First	6.28 (0.91)	6.47 (0.68)	6.34 (1.03)	6.32 (0.94)	6.45 (0.66)	5.40 (0.91)	1.66 (0.87)	2.81 (1.10)			
	Second	6.47 (0.81)	6.20 (0.85)	3.85 (1.86)	4.81 (1.55)	6.60 (0.68)	6.18 (0.98)	4.84 (1.48)	5.40 (1.26)			
**Medical care quality**										268.70 (7, 858)	<.001	0.687
	First	6.32 (0.78)	6.46 (0.69)	6.43 (0.81)	6.35 (0.87)	6.50 (0.65)	5.54 (0.85)	1.71 (1.05)	2.71 (1.14)			
	Second	6.49 (0.76)	6.23 (0.82)	3.79 (1.89)	4.76 (1.62)	6.59 (0.70)	6.26 (0.87)	4.90 (1.52)	5.41 (1.19)			

### Research Question 1

RQ1 was interested in determining how the information viewing condition was related to whether the participants would decide to make an appointment with the provider. A chi-square analysis resulted in a significant finding: χ^2^_7_=216.1 (n=866) and *P*<.001. In only two instances (ie, both conditions where participants saw all negative reviews) did the number of people who indicated not wanting to make an appointment with the provider outnumber those who would. However, even in these two conditions, a large number of participants (95/220, 43.2%) indicated that they would make an appointment.

### Research Question 2

This RQ sought to understand what information influenced people the most regarding their decision to select the provider. A chi-square analysis resulted in a significant finding: χ^2^_14_=288.5 (n=866) and *P*<.001. In only one condition (ie, video first, then all negative comments), the participants reported that the patients’ ratings had the most significant influence. When the participants were exposed to two negative comments or all negative comments first, the video that was viewed influenced the participants more (see Table 3). In conditions where there was agreement in the content that was viewed (ie, reviews predominantly matched the pleasantness of the provider viewed in the video), participants indicated that a combination of the ratings and video were equally influential.

**Table 3:** Participants’ decision making by condition.

Participant decision		Condition, n								Row total
		Video first, then...reviews				...Reviews first, then video				
		All positive (n=105)	Two-third positive (n=101)	All negative (n=106)	Two-third negative (n=101)	All positive (n=126)	Two-third positive (n=100)	All negative (n=114)	Two-third negative (n=113)	
**Participant wants to make an appointment**										
	Yes	98	94	39	59	119	94	56	86	645
	No	7	7	67	42	7	6	58	27	221
**Participant was most influenced by**										
	Video/biography	28	38	32	44	30	54	52	71	349
	Patients’ ratings	5	2	58	34	8	2	39	16	164
	Video/biography and patients’ ratings	72	61	16	23	88	44	23	26	353

### Research Question 3

To more thoroughly investigate why participants exposed to all negative reviews would, or would not, choose to visit the provider, a content analysis of the participants’ open-ended responses was conducted. The two researchers utilized a thematic analysis approach [41] where both researchers independently read all 220 open-ended responses for why participants decided to either want to make, or not make, an appointment with the provider. General themes were developed into a formal coding scheme. Both researchers independently coded the responses with a high level of initial overall agreement (kappa for each category >0.7) and then met to resolve disagreements until 100% agreement was reached. The following themes emerged.

#### Participants Who Would Make an Appointment

A total of 95 people who were exposed to all negative comments mentioned that they would make an appointment with the provider. The following four relevant themes emerged from these participants’ rationales. See Table 4 for all frequencies.

**Table 4:** Rationales for selection of participants exposed to all negative reviews.

Reasoning behind the decision		Participants, n (%)	Example comments
**Would visit the provider (n=95)**			
	Personality of the provider	50 (52.6)	“she seemed genuine” “seems very nice and caring” “she seemed very warm”
	Do not trust reviews	50 (52.6)	“many people online can be dishonest about their visit” “I don’t pay attention to reviews about people, products yes. Some people just grate on each other.”
	Video made the difference	47 (49.5)	“the video tells all” “I liked the way she was in the video”
	Expertise	44 (46.3)	“she seems very competent” “she has experience”
	Other	10 (10.5)	“she sounded like the kind of doctor I would want”
**Would not visit the provider (n=125)**			
	Reviews were bad	113 (90.4)	“the reviews she has were all bad” “I think past patient reviews say a lot and all of hers were negative”
	Did not like the providers’ communication style in the video	12 (9.6)	“I thought her communication was poor in the video” “her tone in the video seemed very cold”
	She is not a doctor	8 (6.4)	“I prefer to see a doctor rather than a nurse practitioner”
	Other	7 (5.6)	“I am male and prefer to speak with a male”

#### Do Not Trust Reviews

A little more than half of the participants who mentioned that they would visit the provider (50/95, 53%) indicated not putting much trust in the patients’ reviews when making decisions. Examples included statements like “I do not always believe what is written by patients/clients/consumers on review sites. I just do not trust the general public when it comes to impartial opinions,” and “I would want to make up my mind on my own instead of relying on the opinions of strangers...There could be a lot of reasons why someone would leave a negative review, sometimes out of spite or because they did not get their way.”

#### Personality of the Provider

A little more than half also indicated that their choice was because of the positive personality characteristics perceived (50/95, 53%). Examples included statements such as “she seemed sincere,” “she seems genuine,” and “she seemed to be very nice, compassionate.”

#### Video Made a Difference

Nearly half of these participants (47/95, 50%) explicitly mentioned the video as a deciding factor. For example, “I would make an appointment with her after seeing the video,” “I am relying on my own judgement from her behavior in the video,” and “I feel like I know much more about what to expect after seeing the video.”

#### Expertise

Just less than half (44/95, 46%) referenced the provider’s expertise as a deciding factor. Perceived expertise was seen in comments such as “she has a long career with good experience,” “she seemed smart, capable,” and “I liked her credentials, her experience.”

Multiple themes could have been present in each statement. For example, 14 responses contained all themes. An example of one of these was “I would make an appointment with her after seeing the video because she seems like a very nice and experienced person. Originally, I thought that she was going to be very rude and unprofessional based on the reviews I had seen. However, I now feel that the reviews were wrong.”

#### Participants Who Would Not Make an Appointment

A total of 125 participants who were exposed to all negative reviews indicated that they did not want to make an appointment with the provider. The following three relevant themes emerged.

#### Reviews Were Bad

Overall, 90.4% (113/125) of these participants indicated that the negative reviews played a deciding factor. Examples include statements such as “I take reviews from people with experience very seriously, and they were all negative,” “I trust patient reviews more,” “based on reviews, I believe those that have seen her, especially when the reviews are so consistent,” and “based on the people that saw her and talked about her I would not care to be involved with her at all.”

#### Did Not Like the Communication Style in the Video

Furthermore, 9.6% (12/125) of these participants also indicated not liking the communication style the provider displayed in the video. For example, “she seemed insincere when interacting with the patient,” “watching the video confirmed [for] me that she is not that friendly,” and “I feel like I can read people well by body language and facial expressions. Based on that alone, the vibe she gives me is still impersonal and not very warm.”

#### She Is Not a Doctor

Overall, 6.4% (8/125) of the participants indicated not wanting to make an appointment because the provider was not a doctor. Examples included statements such as “I would prefer an M.D.,” “nurse practitioners are not doctors,” and “I prefer to see a doctor rather than a nurse practitioner.”

## Discussion

### Principal Findings

This study strove to determine how provider-controlled content (ie, providers’ online biographies/videos) and uncontrolled content (ie, online patient reviews) interact to influence patients’ attitudes and decision-making processes. The findings revealed that the initial deleterious effects of viewing negative patient comments can be significantly reversed when provided with a realistic preview of the provider through a short video introduction. In other words, any initially negative attitudes toward the provider after viewing negative reviews did not persist after viewing the provider’s video. Although the participants’ attitudes did not reach the same heights as when people only viewed positive comments, this research does show health care organizations that hosting videos of providers on their websites can provide a significant buffering effect to negative comments that might exist online via third-party rating websites.

The only conditions in which the provider’s video had the least amount of impact on choosing to visit the provider was when participants viewed comments that comprised all negative reviews. However, even in these conditions, just under half of the participants (43%) chose to go against the reviews and indicated wanting to make an appointment. In these instances, about half of the participants indicated that the video played an important role with one participant stating that “the reviews must have been fake because she seems genuine, compassionate, and capable.” Prior research supports the claim that patients seek providers whom patients perceive as having good interpersonal skills [42]. Therefore, providing prospective patients with videos can offer a realistic preview into how an interaction with the provider might unfold, allowing patients to make up their own minds even in the face of contradictory reviews.

### Extending Expectancy Violations Theory

Although expectancy violations theory was originally applied to nonverbal behaviors [10], it was later extended to verbal behaviors in the context of face-to-face interactions [9]. Since then, the theory has been applied in computer-mediated [43] and mass-mediated settings [44] as well as health settings such as health campaigns [45] and patients’ expectations for communication with a physician [46]. This study breaks new ground in the application of expectancy violations theory by incorporating the comparison between multiple sources—a provider’s online video introduction and online patient ratings—to demonstrate how providers’ videos can be used to generate positive expectancy violations in the case of negative provider reviews.

### Limitations and Future Directions

The first limitation of this study was that only a positively perceived provider was utilized in this study, allowing for only positive expectancy violations to take place. However, given that the majority of patient reviews found online are positive [6], providers may exist who could induce negative expectancy violations. In other words, future studies may want to provide conditions where a provider receives positive reviews but appears grumpy and gruff in his or her video. Future studies may also want to vary video length to determine how short of a video (ie, how thin of a slice) could work to still be effective at providing a significant buffering effect to negative comments.

This study also only tested reviews appearing on the extremes with comments being bipolar opposites of one another (eg, five stars or one star). Future studies might want to test the effects of reviews that are more middle of the road, ie, combinations of two, three, and four stars, and how these ratings interact with providers’ online biographical content provided by health care organizations. In addition, in this study, the content of the reviews focused on the provider’s communication. Future research may also want to include comments discussing other qualities of the provider (eg, credentials and technical competence).

### Conclusions

The rise of health care consumerism today means that patients are shopping around for providers more than ever before. A recent report of top health industry issues reveals that more than three-quarters of consumers desire a “menu of care options offered by multiple providers, allowing them to choose care from local providers or virtual care from specialists across the country.” [47] As Perrault and Hildenbrand [2] found, two of the most popular sources patients are using to seek this information are providers’ own online biographies and patient reviews online. Therefore, continuing to only research the impact of each of these channels in isolation on patients’ perceptions will only provide limited conclusions.

More importantly, recent media reports indicate that fake reviews of medical providers are on the rise, possibly attributing them to competing offices, disgruntled former employees, or even image repair companies seeking to make a profit [48]. Doctors can even pay large sums of money to hide negative reviews or hire reputation management firms [49,50]. However, what this research found is that there is a much less expensive solution to combat potentially false negative reviews online—offering a realistic preview of the provider through short video introductions on providers’ own profiles. As this study revealed, providing a video to participants initially exposed to primarily negative reviews can produce a positive expectancy violation and turn initially negative perceptions into positive ones. Most importantly, more than 40% of the participants exposed to all negative reviews indicated wanting to choose to visit the provider anyway, with many of those indicating that the video helped in making this decision.

In the increasingly competitive world of health care, if providers continue to only offer prospective patients limited information about themselves (eg, text biographies that only provide credentials)—information that is currently the norm [16], patients’ perceptions may become overly clouded by reviews that they read online, whether they are genuine or not. However, if health care organizations decide to offer patients videos that can actually showcase how providers communicate, these organizations may just find that patients are willing to trust their own intuitions. As one participant stated, “you can’t fake nice.”

## Appendix

Multimedia Appendix 1 Video and biography presentation.

Multimedia Appendix 2 Provider ratings (all positive reviews).

## Abbreviations

ANOVA: analysis of variance
PCC: patient-centered care
RQ: research question
