# Supplementary figures and images for: The Buffering Effect of Health Care Provider Video Biographies When Viewed in Combination With Negative Reviews: “You Can’t Fake Nice”
Source: J Med Internet Res. 2020 Apr 14;22(4):e16635. doi: 10.2196/16635 (PMC7189248; doi:10.2196/16635)

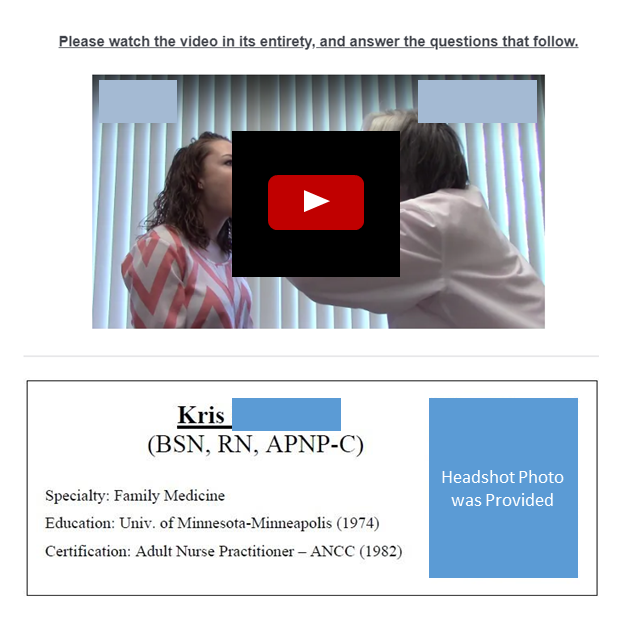

Supplement: Multimedia Appendix 1 [file jmir_v22i4e16635_app1.png]

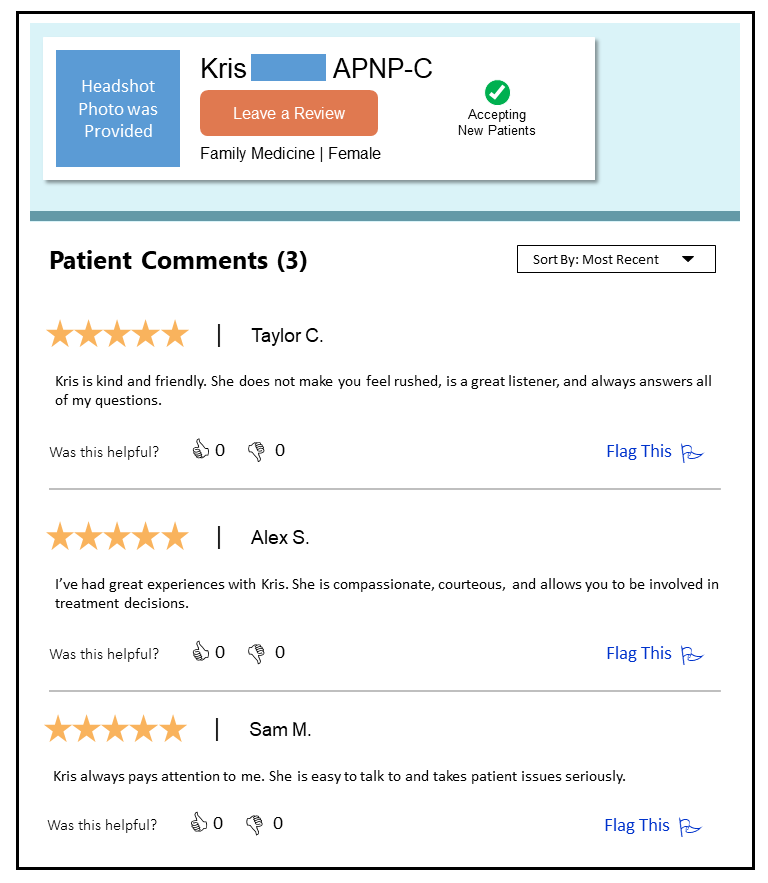

Supplement: Multimedia Appendix 2 [file jmir_v22i4e16635_app2.png]
